# Why Do So Many Calves Die on Modern Dairy Farms and What Can We Do about Calf Welfare in the Future?

**DOI:** 10.3390/ani3041036

**Published:** 2013-11-04

**Authors:** John F. Mee

**Affiliations:** Animal and Bioscience Research Department, Animal & Grassland Research and Innovation Centre, Teagasc, Moorepark, Fermoy, Co. Cork, Ireland; E-Mail: john.mee@teagasc.ie; Tel.: +353-25-42387

**Keywords:** bovine perinatal mortality, epidemiology, aetiology, animal welfare, re-prioritization

## Abstract

**Simple Summary:**

High calf loss rates are an international welfare problem though this is often not recognised. These loss rates have increased in recent years. Improvement in calf survival rates is dependent upon re-prioritization of this problem relative to other animal health and welfare issues and creation of awareness of this prioritization. Once the problem is recognised action needs to be taken at national and at farm levels, specifically on problem farms. Data recording, research, breeding, veterinary, extension and farmer organisations all have a role to play in improving bovine neonatal survival and hence improving animal welfare in the future.

**Abstract:**

Poor bovine neonatal survival rates are an international animal welfare issue. The key modifiable risk factors associated with such loss are age at first calving in primiparae, calf breed, gender and gestation length and calving management. The primary causes of mortality in the perinatal period are calving problems, in particular dystocia, defined as both difficult and abnormal calvings. Calf loss rates are rising on modern dairy farms in many countries internationally. High calf loss rates are often not recognised at national or at farm-level; recording needs to be improved. Improving bovine neonatal survival requires re-prioritization of this issue. Stakeholders need to be made cognisant of this prioritization. Actions to effect change need to occur at both national and farm-levels. National-level actions need firstly to address raising awareness of the issue. Farm-level actions need to focus on identifiable problem farms through targeted surveillance. Application of existing knowledge to alter modifiable risk factors is the key to improving calf welfare in the future. Research also has a role to play in filling knowledge gaps in particular about the ‘unexplained stillbirth’.

## 1. Introduction

Perinatal mortality may be defined as death of the perinate prior to, during or within 48 hours of calving, following a gestation period of at least 260 days, irrespective of the cause of death or the circumstances related to calving. The perinatal period is the most hazardous in the life of all animals. Approximately 75% of perinatal mortality occurs within one hour of calving with the remainder occurring either pre- (10%) or post-partum (15%). Some 90% of calves, which die in the perinatal period, were alive at the start of calving and so much of this loss is a preventable welfare problem [1].

Traditionally perinatal calf mortality was considered an indicator of management quality but was ‘*a welfare concern that seemed to be all but ignored*’ on dairy farms [2]. However, it is now considered the ‘*most crucial indicator of welfare level*’ [3]. Perinatal calf mortality rates are one of the most commonly used population-level welfare indices on dairy farms today [4,5,6] and calving management and care of the newborn calf are considered critical areas of herd management affecting calf welfare [7]. At the individual calf level, pain and injury associated with a difficult calving is deemed by veterinary practitioners to be a welfare issue requiring therapeutic intervention [8,9]. However, the evidence from neurobiology and electroencephalography on compromised perinatal welfare is less clear. In a seminal paper on the welfare implications of neonatal problems it has been argued that the key subjective noxious experiences may be theoretically and provisionally ranked in ascending order as breathlessness, hypothermia, hunger, sickness and pain [10], and these may also occur concurrently. Even though these problems may result in perinatal mortality this may or may not be a welfare problem *per se* but may an animal rights problem. For example, the pain experienced by a calf due to parturient traumatic injuries (e.g., fractured leg, mandible, ribs, spine, ruptured internal organs or diaphragm or severe internal haemorrhage) or prolonged or forceful traction suffered during and immediately after calving from forced extraction (with a mechanical calving aid) is considered a serious welfare insult [10]. In order to experience pain the foetus must be conscious which is accepted by some [11] but not by others [10]. Thus, a calf which dies due to anoxia before or during calving does not achieve a conscious state and so although they can generate a physiochemical stress response their welfare is technically uncompromised. The impact of each individual cause of calf mortality on animal welfare is discussed in the section on *Cause of Death* (*COD*). From an animal rights perspective the death of an animal in the perinatal period clearly deprives it of its right to a normal life expectancy (‘*to lead a life appropriate for their kind*’). An adjacent dilemma exists regarding the fate of calves, in particular male dairy calves, which survive the perinatal period but in some industries internationally are euthanized prematurely due to their low economic value. Irrespective of this welfare ranking, all perinates merit attention and ‘*a distinction based on ranked suffering would rarely, if ever, be made when devising preventative strategies and when treating affected newborns individually*’ [10]. Thus, there is an apparent conflict between the perceived importance of the consumer’s perception of compromised perinatal welfare or the animal’s rights and the science underpinning foetal consciousness and ability to suffer poor welfare.

So many calves die because their death is not prioritized, not only as an economical but also as a welfare problem [12]. In the absence of such prioritization an intention-behaviour gap exists. This may be due to the asymmetrical perception of the issue by farmers and their veterinarians on the one hand and by supermarkets and their consumers on the other hand. There is a danger that while on farm high perinatal mortality rates may not be recognised or are accepted as part of farming (‘*where there are livestock there are deadstock’*), within the non-agricultural milieu such losses are increasingly unacceptable. Hence, there is a need for both pressure through welfare audits to improve animal welfare, including that associated with perinatal mortality, but also, a heightened awareness amongst farmers and their veterinarians of the long-term consequences of non-prioritization of perinatal mortality in an age where retail oligopolies dictate the economic future of their clients. Examples of national initiatives to increase hazard perception for calf mortality amongst the relevant agricultural stakeholders include the CalfCare technical working group in Ireland (www.animalhealthireland.ie) and the ‘Stop the Loss’ campaign in the UK (www.nationalyoungstock.co.uk). The risk factors associated with high calf losses are well documented. Yet, in recent years calf loss rates have increased in many countries internationally [13,14,15,16]. The reason for the lack of improvements in neonatal survival stems from de-prioritisation of the issue relative to other animal health and welfare concerns [12]. This has resulted in less funding of research work in perinatology in comparison with that for the well-documented decline in dairy cow ‘fertility’, of which it is an adjacent problem. Consequently there has been downstream atrophy of knowledge metastasis through extension and implementation programmes. There are knowledge gaps constraining progress towards improved neonatal survival requiring more transdisciplinary research including the ‘omic’ technologies. The neologism omic refers here to new fields of study in biology, such as genomics, proteomics or metabolomics. An example of how omic research can be used to reduce perinatal mortality is the recent discovery of an association between single nucleotide polymorphisims in the leptin gene and bovine perinatal mortality; mortality was two-fold higher for Holstein heifers with a particular leptin genotype which is associated with placental growth [17]. The potential exists to use this genetic information as a tool to aid in selection for reduced perinatal mortality. However, re-prioritisation of neonatal survival as an important animal welfare deficiency signal, often by the retail arm of the food industry [5], and better communication of existing knowledge are of greater practical importance in reversing current trends.

The answer to the titular question, ‘what can we do about calf welfare in the future?’ is—in theory—quite a lot, but the inconvenient truth is in practice often, limited progress. This dichotomous answer hints at the enigmatic discord between what is theoretically possible and what actually occurs in practice. Though this view may conflict with perceived thinking, evidence for this divergence can be found in the disparity between bovine neonatal survival rates (in the first two days of life) internationally and between results from research studies and farm-level data. For example, dairy calf neonatal mortality rates in some countries, e.g., Norway, are amongst the lowest in the world [18], in contrast to those in many North American Holstein-Friesian-dominated dairy industries [16]. Whereas experimental and observational studies have identified critical risk factors for improved neonatal survival [19,20,21], the results from such studies are not always replicated at farm level. In fact the reverse has occurred in recent years with a decrease in bovine neonatal survival rates reported in the peer-review literature from many countries around the world [13,14,15,16].

By reviewing the epidemiology and aetiology of dairy calf mortality, this paper at first considers the most important reasons why so many calves die and secondly proposes approaches to reversing current trends in the future. All this is addressed in order to increase awareness of calf mortality as a priority of animal welfare on modern dairy farms.

## 2. Why Do So Many Calves Die?

### 2.1. Epidemiology of Bovine Perinatal Mortality

#### 2.1.1. Incidence of Perinatal Mortality

Currently, the average incidence of perinatal mortality in cows and heifers varies between 2 and 20% across dairy industries internationally with the majority of countries between 5 and 8% (Table 1). The variation between national agricultural statistical data averages reflects differences in definitions of perinatal mortality but, more importantly, emphasises the differences between those countries which have practised a long-term policy of genetic selection against undesirable functional traits (e.g., Norway and Sweden) and those which have pursued single trait selection policies (e.g., Canada and the USA) and associated dairy breed differences [22]. The most worrying incidence data are those from the US, as these genetics are exported around the world and could influence rates in almost all dairy industries worldwide [23]. The data in Table 1 also highlight the lack of conformity in recording of perinatal mortality and the definitions used to describe it; the need to standardise such definitions is self-evident when attempting to make valid international comparisons. 

These average national figures obscure the fact that herd-level statistics follow a right skewed distribution where most herds have none or minimal losses but some herds have 20% to 30% perinatal mortality [19,24]. Thus, even within countries with a relatively low incidence rate, problem herds exist. Despite the best efforts of farmers and their veterinarians to manage calving and newborn calves successfully, perinatal mortality can be a perennial problem on some farms, yet only occur sporadically on others. Currently there is little research on the causes of this wide inter-herd variation in stillbirth rates and why certain herds have persistent problems and others do not [25]; well-designed transdisciplinary studies are warranted.

**Table 1:** Incidence of perinatal calf mortality in dairy heifers and cows in 20 countries internationally (2000–2011).

Country	Breed of dam	Heifers (%)	Heifers and cows (%)	Definition of calf mortality	Reference
Australia	HF	10.8	5.1	Death within 48 hours of a singleton calving	[26]
Austria	HF	8.7	5.9	Death within 48 hours of calving	[27]
Canada	HF	9.0	9.6 ^a^	Dead at birth	[28]
Denmark	HF	9.0	NR ^b^	Death within 24 hours of calving	[14]
Germany	HF & HFx BP	NR	9.3	Death within 24 hours of calving	[29]
Iceland	In	23.0	15.0	Stillbirth	[30]
India	Je	NR	3.8	Foetal death	[31]
Israel	HF	7.2	5.0	Death within 24 hours of calving	[32]
Iran	HF	4.3	3.5	Death within 1 hour of calving	[33]
Ireland	HF	7.7	4.3	Death within 24 hours of calving	[24]
France	Dairy	NR	7.4	Death within 48 hours of calving	[34]
Hungary	HF	NR	7.7	Death within 24 hours of calving	[21]
The Netherlands	HF	16.6	5.0	Death within 24 hours of a singleton calving	[15]
New Zealand	HF Je and their crosses	7.4	7.2	Death within 48 hours of calving excluding inductions.	[35]
Norway	NR	3.0	2.0	Death within 24 hours of calving	[18]
Poland	HF	8.1	6.7	Death within 24 hours of calving	[36]
Sweden	SR	3.6	2.5 ^a^	Death within 24 hours of a singleton calving	[37]
Switzerland	Dairy, Beef & Crossbreeds	5.9	2.4	Death within 24 hours of calving	[38]
UK	HF	12.1	7.9	Death within 48 hours of a singleton calving	[39]
USA	HF	12.1	8.0	Dead at birth	[16]

^a^ cows only, ^b^ not recorded, BP = Blackpied, HF = Holstein-Friesian, Je = Jersey, Mo = Montbeliarde, No = Normande, NR = Norwegian Red, SW = Swedish Red.

#### 2.1.2. Temporal Trends in Perinatal Mortality

Recent published studies in Denmark [14], The Netherlands [15], North America [16] and Sweden [13] indicate that the prevalence of perinatal mortality is increasing, particularly in Holstein heifers. Much of this increase has been attributed to North American Holstein introgression, or introduction of particular Holstein sires’ genes, into indigenous cattle populations. The resultant calves have a longer gestation length, are larger and heavier at birth, suffer more difficult calving and consequently are at greater risk of perinatal mortality [13,40]. Dutch data suggest half of the increase in stillbirth in first calvers is attributable to genetics and the rest to changes in management [15]. Recently it was reported that stillbirth rates had changed very little on UK dairy farms in the past 10 years [39], and at 8% now accounted for over twice as much calf mortality as neonatal losses. Unfortunately, there are no long-term longitudinal necropsy studies which explain which particular causes of perinatal mortality have increased over time or whether there has been an increased incidence of all cause mortality. This is a knowledge gap requiring research if we are to address future changes in loss rates and causes. 

#### 2.1.3. Risk Factors for Perinatal Mortality

The majority of perinatal mortality has been attributed directly to difficult calving particularly in heifers, which frequently require assistance at calving. Parity has been shown to be the best predictor variable for perinatal mortality followed in heifers by difficult calving and in older cows by difficult calving and gestation length [41]. Other significant animal-level factors, also common to difficult calving, include age at first calving, particularly in heifers less than 24 months old [30], twinning [16], foetal gender [13], shorter or longer gestation length [41] and sire predicted transmitting ability (PTA) for perinatal mortality [24]. In recent years, the interplay between genotypic and environmental risk factors has received more scientific attention with the identification of modifiable and non-modifiable risk factors for perinatal mortality [20]. Crossbreeding studies have now illustrated the differences in perinatal mortality between different dairy and dual-purpose breeds [22]. The increase in perinatal mortality with increasing proportion of Holstein-Friesian genes in both the calf and in the dam has been demonstrated [40]. In addition, the role of inbreeding as a significant risk factor for perinatal mortality has only recently been documented, and though the effects are small and mainly confined to heifers, they were consistently unfavourable [42]. 

Significant herd-level risk factors for perinatal mortality include herd [16], year [43], season of calving [43], larger (>20 cows) herd size [44] and calving management [19]. While deficiencies of micro-nutrients (iodine, selenium, copper and zinc) have been associated with high stillbirth rates [45,46], results from randomised clinical trials have not always supported a causal relationship [47,48]. Excess body condition prior to calving, particularly in heifers [49], has been associated with reduced appetite as calving approaches with resultant mobilisation of fat reserves; also it may reduce magnesium availability, and the ensuing sub-clinical hypocalcaemia could produce uterine atony which is observed clinically as ‘slow calving syndrome’ where foetal death occurs in the absence of difficult calving (non-visible dystocia) [50].

Management of calving plays a critical role in perinatal mortality in dairy or beef herds [1]. For example, increased duration of second stage calving beyond two hours, poor abdominal contractions, use of mechanical calf pullers and changes in the calving supervision all increase significantly the risk of perinatal mortality [51]. 

In addition to these accepted risk factors, there is now evidence that an increasing proportion of perinatal mortality occurs at unassisted calvings where placental dysfunction and low birth weight may be causative factors [30,52]. Idiopathic stillbirth or weak calf syndrome is particularly associated with heifer calvings. 

### 2.2. Time of Death (TOD)

The causes of calf loss, as opposed to the risk factors associated with such losses, are best diagnosed from necropsy examination coupled with a clinical anamnesis and supportive laboratory analyses as necessary.

In human paediatric medicine an autopsy is considered valuable in determining the time and the aetiology of perinatal mortality. However, in veterinary medicine necropsy of the perinate is often seen as a low yield diagnostic technique by farmers, veterinary practitioners and by veterinary pathologists. This view discourages both farmers and veterinary practitioners from submitting carcasses or samples from carcasses for examination, hence the very low submission rates [53]. However, diagnosis rates varying between 52% and 96% have been reported in recently published studies (Table 2). Necropsy examination can determine both the time of and the cause of death. Thus, necropsy examination is critical to the understanding of why do so many calves die [54].

The timing of perinatal mortality may be determined from the medical history provided by the farm personnel, the degree of pulmonary atelectasis, the degree of autolysis of the carcass or by evidence of postnatal survival, e.g., umbilical thrombi or worn foetal hooves. 

#### 2.2.1. Preparturient *vs.* Parturient Mortality

The degree of carcass autolysis has been used to estimate the duration of retention *in utero* following foetal death. These estimates are based on sterile foetal autolysis in calves [55], lambs [56,57,58] and in piglets [59] which may differ from the sequence in the non-sterile foetus. Similar findings have been reported for human foetuses [60,61].

#### 2.2.2. Parturient *vs.* Post-Parturient Mortality

Parturient mortality can be differentiated from post-parturient mortality by the farm personnel history or by evidence of postnatal survival, e.g., the degree of pulmonary atelectasis, umbilical thrombi or worn foetal hooves. Where the calving is unobserved the pathologist must rely on the evidence from the carcass to estimate TOD. Calves, which were born alive have partially or fully inflated lungs [55]. The degree of atelectasis has been recorded in a limited number of studies. In most cases, the authors referred to ‘lung expansion’, functional lungs’, inflated lungs’ or ‘the lungs float in water’ as the criteria for the presence or absence or degree of atelectasis. In the most detailed description of the degree of atelectasis in 309 calves which died within approximately 10 minutes of birth complete atelectasis, partial atelectasis and no atelectasis was found in 44%, 24% and 32% of calves, respectively [62]. Stillborn calves do not have large (>4 mm diameter) umbilical thrombi, which are present in calves, which were born alive but died [55]. The presence of umbilical thrombi indicates a functional heart in the calf after calving. However, small thrombi may occasionally be found in calves, which die during or immediately after prolonged calving. If the calf has walked the palmar/plantar surface of the hooves, they will be worn off indicating post-natal survival [58].

### 2.3. Cause of Death (COD)

The major causes of bovine perinatal mortality as described in recent necropsy studies internationally are dystocia (approximately 35%) and anoxia (approximately 30%), to a much lesser extent, other causes (approximately 15%), infections (approximately 5%) and congenital defects (approximately 5%), (Table 2). On average, some 25% of cases have no diagnosed cause but this varies between approximately 5% and 50% between studies (Table 2). The variation in the proportions of necropsy-diagnosed causes of death reflects variations in the causative risk factors but also variations in diagnostic definitions and the number and selection criteria for calves and herds examined. The impact of cause of death on animal welfare is dependent upon the characteristics of the individual circumstances preceding each individual cause of death as outlined hereunder for each COD.

**Table 2:** Necropsy-diagnosed causes of death (%) for calves dying in the neonatal period internationally (2000–2011).

Country	Calves (No.)	Dystocia	Anoxia	Congenital defects	Infection	Other	Unknown	Reference
Canada *	560	40.2	NR **	4.3	2.9	31	21.6	[63]
Finland	148	43	***	10	10	8	29	[64]
Iceland	129	34	37	NR	12	13	3.9	[65]
Ireland	680	27	6	3	3	49	12	[66]
Netherlands	180	***	41	4.4	6.6	5.6	48	[67]
Sweden	76	46.1	NR	5.3	2.6	10.5	35.5	[52]
USA	60	25	28.5	3.3	5	6.6	31.6	[68]

* Beef calves; all others are dairy calves, ** NR = not recorded, *** Anoxic and difficult calving lesions combined.

#### 2.3.1. Dystocia

Traumatic lesions found in stillborn calves associated with dystocia include fractured and dislocated ribs, fractured spine, fractured legs, fractured mandible, diaphragmatic tears or hernia, hepatic rupture, renal haematoma, subcutaneous haemorrhages, bruising or oedema around the neck, subdural haemorrhages, internal haemorrhage, and collapsed trachea [69]. The most common lesions recorded are fractures of the ribs or the spine. The trauma associated with dystocic mortality is clearly a serious animal welfare issue arising from the pain and suffering the calf endures prior to death.

#### 2.3.2. Anoxia

Anoxic lesions, often found following clinical dystocia and ‘non-clinical dystocia’ (clinically undetectable prolonged or abnormal stage one or two of calving), include pulmonary atelectasis, subserosal haemorrhages (pleural, tracheal, scleral, epicardial, endocardial), organ congestion (liver, kidneys, conjunctiva, meninges), meconium aspiration syndrome (MAS) and meconium staining or passage [69]. Unfortunately, calves dying following acute anoxia often have unremarkable gross pathological findings. Prolonged hypoxia may cause more pronounced lesions [70]. Mortality due to acute anoxia where the foetus has not achieved a conscious state is not an animal welfare issue. Even though chronic hypoxia may result in pronounced physiochemical stress and grossly visible extensive lesions upon necropsy, if the foetus is not conscious during these life-threatening changes, technically this does not impact upon its welfare.

#### 2.3.3. Infections

Unlike abortions where infections constitute the major proportion of diagnosed causes, in perinatal mortality infections are a minor diagnosed cause varying between 3% and 15% between published studies (Table 2). However, the infectious agents associated with abortion are the same ones associated with perinatal mortality; *Truperella pyogenes*, *Bacillus* spp, bovine viral diarrhoea virus, *Brucella abortus*, *Coxiella burnetii*, fungi, *Leptospira hardjo*, *Neospora caninum*, *Pasteurella multocida* and *Salmonella dublin*. In addition, inflammatory lesions indicative of bacterial infection (e.g., bronchopneumonia, encephalitis) are used as diagnostic criteria for infection as a cause of death in cases of perinatal mortality and are often ranked as the most commonly detected criterion. *In utero* mortality caused by pathogenic infections do not compromise animal welfare, however, the death of perinates born following *in utero*, transvaginal or postnatal infections which subsequently suffer from the consequent inflammatory lesions, e.g., omphalophlebitis, pleuro-pneumonia, peritonitis, is an animal welfare issue.

#### 2.3.4. Congenital Defects

Congenital defects may be defined as any defect in the foetus present at birth. However, anatomical abnormalities are those most commonly diagnosed by veterinary practitioners and non-specialist veterinary diagnostic laboratories. The incidence and types of congenital defects are highly variable depending primarily on the survey methodology. Hence, the number and types of cases submitted to veterinary institutions (research labs, routine diagnostic labs, and veterinary faculties) may differ greatly from those actually occurring on farms, and observed by veterinary practitioners, which are not submitted. This submission bias may result in fewer but more severe cases being submitted than non-submitted. Thus, in passive surveillance surveys the incidence may be quiet low (*ca.* 5% of examined calves, Table 2) and consist of severe cases while in active surveillance surveys the incidence can be quiet high (*ca.* 20%) [71], but include many non-lethal cases. 

Defects may be lethal, sublethal (economically lethal) or non-lethal with the former predominating. Defects may also be single or multiple with the former predominating. The majority of multiple defects are lethal. Congenital defects found in cases of perinatal mortality include atresia of the intestines, arthrogryposis, cerebellar hypoplasia, cleft palate, hydrocephalus, omphalocoele, and ventricular septal defects. Detection of multiple lethal defects is not difficult upon routine necropsy examination as most are easily visible (e.g., schistosomus reflexus) [69]. However, diagnosis of some single defects requires careful examination of all organs (e.g., unilobar thyroid gland) or more detailed examination not routinely carried out in veterinary practice (e.g., craniotomy for moderate hydrocephalus). The common putative causes of congenital defects, genetic mutations, environmental teratogens, pharmaceutical teratogens and infectious teratogens are difficult to definitively diagnose in veterinary diagnostic laboratories or in veterinary practice. Increasingly genomic and proteomic technologies are being developed which will provide diagnostic tests to determine whether there is a hereditary basis to many defects. The impact of congenital defects on animal welfare is dependent upon the consequences of the defect. For example, a calf with an economically lethal defect (e.g., vestigial limb), which is humanely euthanized does not suffer compromised welfare. However, a calf born with an intestinal atresia necessitating calving assistance which causes rupture of the proximal distended intestine, inguinal herniation and leakage of the intestinal contents into the peritoneal cavity has compromised animal welfare. Similarly, if a calf with such an atresia is not diagnosed as having the condition and is continually fed or force-fed milk it will suffer from the pain of a grossly distended gastrointestinal tract for days before its inevitable death. 

#### 2.3.5. Omphalorrhagia

Omphalorrhagia may be defined as bleeding from one or both umbilical arteries. With internal omphalorrhagia the arteries retract into the abdomen but do not constrict completely. Cases vary in severity from minor perivascular haematoma to extensive haemoperitoneum and intra-abdominal coagulum formation in the absence of other sources of haemorrhage. Severe cases predominantly occur in full term bovine foetuses though they have been recorded in aborted foetuses. Affected calves tend to die between 1 and 48 hours after birth. Such calves are generally found dead without premonitory clinical signs. The most common presenting sign is conjunctival pallor. There are no published data on the prevalence of this condition in newborn calves. While the aetiology of this condition is largely unknown many hypotheses have been proposed. These include rapid severance of the umbilical cord as may occur following Caesarean section births rather than stretching and gradual separation, prematurity, the Chediak-Higashi syndrome, BVDv thrombocytopenia, mycotoxins, factor XI genetic defect and maternal injury through stepping on the calf. Farmer treatment of affected cases by ligating the cord is ineffectual as the bleeding is internal. Veterinary practitioners have used blood transfusions and surgical ligation with variable success. The welfare of calves affected by omphalorrhagia is clearly compromised, as they tend to survive for hours or days while continuing to haemorrhage internally and become more anaemic.

#### 2.3.6. Premature Placental Separation (PPS)

In the cow the foetal membranes are normally expelled between 30 minutes and 8 hours after stage two of calving. While premature placental separation (PPS) is a well-recognised condition in the mare, there is a paucity of literature on the condition in cattle. Premature placental separation has been associated with ‘weak calf syndrome’ in heifers [72]. It has been associated with premature birth [73] and maldisposition [74]. Anecdotally, pharmacological induction of parturition, excessive selenium supplementation and subclinical hypocalcaemia have also been implicated. It is considered, where recorded, as a minor cause of perinatal mortality. Calves, which die following PPS do so due to anoxia or haemorrhage *in utero* or during calving and as such their welfare is not compromised.

#### 2.3.7. Trace Element Disorders

Classical deficiency of trace elements, for example iodine [75] and selenium [46], is still associated with high perinatal mortality rates in individual herds, particularly in heifers. Associations have also been made between herd blood copper, zinc and selenium status and perinatal mortality [45]. Respiratory distress syndrome (RDS) in calves has conventionally been associated with prematurity. However, recent research indicates that RDS in mature Belgian Blue calves may be associated with trace element deficiency-induced surfactant insufficiency; specifically, deficiencies of selenium, copper, zinc and iodine [76]. The proportion of perinatal mortality attributable to iodine imbalance is variable in published studies reflecting differences in animal husbandry and diagnostic criteria. Trace element deficiency-induced RDS directly impacts animal welfare as such calves survive after calving but have great difficulty in breathing and, even if diagnosed and treated, many die.

#### 2.3.8. Other Causes

Perinatal mortality following eutocia may be associated with intrauterine growth retardation (IUGR), prematurity with surfactant deficiency, dysmaturity, twins, placental dysfunction or sire-specific genetic weakness. Prolonged stage one, prolonged stage two with uterine atony, nitrate toxicity and accidents may also contribute to eutocic stillbirth. While the circumstances of each calf death due to these various causes will vary widely, those with the greatest impact on animal welfare are live-born premature calves and calves which suffer prolonged stage one or two and are born with severe dyspnoea which subsequently die. In addition, perinates which suffer fatal accidents after calving, such as severely fractured limbs following entrapment in automatic passage scrapers in cubicle houses or aspiration pneumonia following improper use of an oro-esophageal feeder for administering colostrum have compromised animal welfare.

#### 2.3.9. The Unexplained Stillbirth

Despite these findings, recent research indicates that the proportion of perinatal mortality in both dairy and beef breeds attributable to difficult calving and other traditionally diagnosed causes of perinatal mortality may be decreasing [52,67]. A recent pilot study in Dutch dairy herds failed to link high perinatal losses with these traditional causes [67]. Recent Swedish research indicates that increased perinatal mortality in Holstein-Friesian heifers cannot be attributed to increases in difficult calving and that calf vitality may be a critical factor [77]. A genetic predisposition has been posited due to the large variation in perinatal mortality in the daughters of different sires. Further investigations suggested placental dysfunction might explain such genetic differences [78]. In addition, the possibility of undetected intra-uterine infection causing chorioamnionitis and foetal mortality cannot be discounted [79].

In many cases the cause is undetermined. Diagnostic rates in veterinary laboratories are often less than 25% indicating the need for a new approach to perinatal loss investigation [66]. Additionally, as the incidence of idiopathic perinatal mortality appears to be increasing there is a need for renewed research focus on this cohort of calves to determine the modifiable risk factors and cause of this syndrome. A clear case definition, intensive anamnestic, clinical and pathological investigation, generation of plausible hypotheses and testing of such tentative diagnoses in designed, prospective, multisite, population-based field trials will lead to a clearer understanding of the causation of this syndrome. The role of evidence-based veterinary medicine (EBVM) here is self-evident. The impact of the unexplained stillbirth on animal welfare is unknown as the circumstances surrounding such deaths are probably highly variable and the degree of suffering is undefined.

## 3. What Can We Do to Reduce Calf Losses in the Future: New Approaches

### 3.1. Re-Prioritization Is Needed

Before significant improvements in perinatal mortality can occur the issue needs to be re-prioritised. By this I mean the stakeholders must view it as a problem worth doing something about. In the absence of this clarity of vision drift will continue. The first issue to address is ‘farm blindness’ whereby high perinatal mortality rates are ‘normalised’ and the farmer becomes blind to the issue [80]. For example, a recent Norwegian study showed that farmers underestimated the incidence of calf diseases by 40% [81] and a Canadian study has shown that farmers can underestimate loss rates by up to 50% and there is a very poor correlation (r = 0.01) between actual and perceived loss rates. In addition, the majority (94%) of farmers did not consider calf mortality to be a problem even though the average loss rate at birth was 8.8% [82]. Behavioural science research is warranted. In addition, there can be significant under-recording of calving problems in national databases [83], partly due to reluctance by farmers to report or discuss the issue with their veterinarian [84]. In North America, highly variable recording of stillbirth between farms has been attributed to the voluntary recording systems in use [85], though the author did not investigate or report variation in recording rates. Hence, reliable recording is central to improved calf survival; “*if you can’t measure it you can’t monitor it*”.

Once the industry recognises the problem they then need to put it in perspective. Recent UK data show that the loss rates in the perinatal period are more than twice as great as those in all other periods of the animal’s life [39]. In addition, there has been enormous effort poured into getting cows in calf but very little effort devoted to getting the calf out alive, without which the former work is nullified. It is perhaps unfortunate that calving is a means, not an aim; dairy farmers get paid to produce milk and not live calves…but this is changing…

### 3.2. Good Animal Welfare Pays

Whether the agricultural stakeholders act to reduce perinatal mortality or not by their own volition, action may be forced on them by the retail multiples. Large international retailers, conscious of the attitudes and opinions of their consumers are beginning to act to encourage their farmer suppliers to improve animal welfare, including perinatal mortality. For example, in 2011 a large UK retailer (Tesco) notified its milk suppliers that under its new welfare code of practice it would be requesting them to record calving performance (including difficult calvings and perinatal mortality) [5]. Presumably they will then act on this information when purchasing milk thus correcting the vision of suppliers with ‘farm blindness’ about the link between good animal welfare (for example lower perinatal mortality) and farm profit. Similarly, another UK retailer (Marks & Spencer) does not accept beef from Belgian Blues because of the high Caesarean rate in purebreds and the welfare image problem that presents to the consumer [86]. Additionally, in Sweden a ‘welfare deficit’ index, which includes stillbirths, has been successfully used to identify dairy herds with poor animal welfare [6].

### 3.3. Raising Awareness

Without proactive national awareness, action will be incoherent or possibly forced upon the industry. There is a critical role for extension in creating awareness and in knowledge metastasis. Raising awareness is predicated upon a national knowledge infrastructure involving field extension officers, veterinary organisations, farmer organisations and farming media support. National awareness campaigns have been successfully used to highlight adjacent problems such as poor cow fertility (In-Calf, Australia and New Zealand) and mastitis (CellCheck, Ireland). Recently (2011), national initiatives have been launched in the UK and in Ireland to highlight and to address the issues surrounding calf health. In the UK the National Youngstock Association has been established as a forum to tackle losses in cattle youngstock. A national calf health campaign (CalfCare) has been launched in Ireland through the national animal health organisation (Animal Health Ireland). This campaign is operated through an expert technical working group which produces stakeholder needs analysis and current practices surveys, peer-review publications [87], technical leaflets, media releases and service provider and farmer conferences and road shows.

### 3.4. Acting Nationally

At the national level stakeholders can improve neonatal survival by (1) creating awareness, (2) funding relevant research, (3) altering their genetic selection policy and (4) by addressing endemic infectious diseases impacting calf health. Genetic tools to improve neonatal survival nationally include composite genetic selection indices, encouraging crossbreeding, reducing inbreeding depression and developing genomic selection for calving/calf trait-associated QTLs. Internationally examples exist where genetic selection to improve neonatal survival has been occurring successfully for decades, e.g., Norway (1978), or has recently been introduced, e.g., Ireland (2005). Currently calving traits account for 10.3% of the overall emphasis in the Irish Economic Breeding Index (EBI) (www.icbf.com). National control programmes for endemic infectious diseases have recently gained significant momentum internationally. For example, a voluntary national BVD eradication programme was launched in the Republic of Ireland in 2012 (www.animalhealthireland.ie), which became mandatory in 2013, which should significantly impact calf health. 

### 3.5. Role of Research

There are a few foci of veterinary scientists active internationally, who will continue to produce advances from breeding to birth contributing to our understanding of bovine neonatology. Likely future breeding developments include the greater use of genomic selection, exploiting the recently mapped bovine genome, to breed for reduced difficult calving and stillbirth using sharper phenotypes and possibly more widespread use of sex-sorted semen. Development of parturient ethograms combined with point-of-care sensor technologies will improve accuracy of prediction of onset of parturition; continuous foetal monitoring during parturition can detect reduced vitality; refinement of current therapeutic protocols will improve resuscitation of compromised perinates; improved periparturient pain management and more standardised necropsy work-up after stillbirth will improve our understanding of perinatal welfare and mortality aetiology, respectively.

Developments in the “omic” technologies will transform cattle breeding in the future, however, the quality of phenomic records (e.g., parity-specific trait observation) may determine the rate of progress in reducing perinatal mortality [88]. Recent developments in cattle breeding indicate that future genetic total merit indices will benefit from adjusting weights to use evaluations for perinatal mortality from primiparae and pluriparae separately [89] and inclusion of a maternal calving ease estimated breeding value (EBV) [90]. Given our current knowledge on the inbreeding coefficient depression of calf survival [42], this coefficient will be included in future breeding programs to reduce the risk of perinatal mortality. The practice of recommending ‘easy calving’ sires for use on heifers has been questioned [91], hence future breeding programs may re-evaluate this practice. In addition, the apparent increase in idiopathic stillbirth may focus future breeding goals away from risk of dystocia alone and towards improved calf vitality. This trend is already proposed for ‘easycare’ sheep breeding to reduce the labour required at lambing time and to reduce ovine perinatal mortality [92].

Scientists and producers have for years attempted to predict accurately the onset of parturition in cattle and hence prevent perinatal mortality and other parturient complications. Until recently, these efforts were hampered by the lack of availability of robust point-of-care sensor technologies suitable for use in the field. In parallel with improvements in biosensors (e.g., www.moominder.ie, www.alerte-velage.fr, www.sisteck.com), recent renewed emphasis on the development of parturient ethograms in order to evaluate the efficacy of such methodologies [93] has stimulated interest in this specialised field. Future developments will include refinement of behaviour and movement sensors, [94,95], utilization of real time image analysis [96] and combination of clinico-physiological indicators into a predictive scoring system [97,98].

Future developments in bovine perinatal monitoring will include more widespread application of blood gas analysis during stage two of calving to predict perinatal acidosis [99], (e.g., using the i-STAT portable analyser), research on novel biomarkers of *in utero* hypoxaemia in calves, (e.g., activin A) [100], measurement of intrapartum foetal oxygen saturation using pulse oximetry [101] and umbilical blood flow using transcutaneous Doppler ultrasonography [102] and further characterization of extrauterine adaptation by monitoring of respiratory function using impulse oscillometry [103]. 

In addition to developments in perinatal monitoring, traditional obstetrical techniques are being looked at anew with preliminary results indicating that veterinary practitioners may need to re-evaluate their approach to traction in cases of bovine dystocia in order to prevent perinatal mortality [104,105]. One area of relative neglect in bovine perinatology research has been cervical physiology approaching parturition. Research work on the biochemical [106] and electromyographic [107] processes involved in final cervical ripening indicate that it may be possible in future to influence this process in order to accelerate parturition where necessary [108]. Another much neglected area of research has been the pain caused by parturition in both the dam and the calf. The recent development of an ease of farrowing score, as an indirect measure of pain, in sows [109], and the availability of analgesics licensed for use in cattle suggest a fruitful area of future research to improve perinatal welfare.

While the development of cloned calves has added application impetus to this research, many of these technologies are currently not directly transferrable to general practice. As with all new developments, transfer from the laboratory to the field involves numerous steps including the commercial viability of the technology, the marketing of new knowledge or technologies by information providers (e.g., using the ‘nudge’ concept), overcoming the intention-behaviour inertia of the end user towards such new developments, and the safety and efficacy of new developments under ‘real world’ conditions internationally. There is a clear role for translational, in particular, behavioural research to transform science into solutions in order to effect real change at individual farm and ultimately cow and calf-levels.

### 3.6. Farm-Level Improvements

At the farm level the application of existing knowledge by practising veterinarians and extension professionals is where most progress can be made. Though the causes of mortality may vary between farms, the causes of loss which warrant particular focus are those which cause greatest loss and which can be reduced, *i.e.*, modifiable causes; parturient deaths caused by dystocia (Table 2). A recent international survey found that veterinary practitioners attributed the incidence of perinatal mortality primarily to the availability, skills and education of farm staff [110]. There was unanimity amongst respondents regarding the action farmers could take to reduce its incidence: better calving management. This included supervision of the late pregnant cow prior to and during calving, use of correct obstetrical techniques, modern calf resuscitation methods and critically, calling the veterinary practitioner at the correct time. When asked how the veterinary practitioner could reduce perinatal mortality rates, respondents agreed that veterinary practitioners needed to focus on client education related to calving management. This is an often-neglected area of a stockman’s education, particularly on large farms, which has been shown to be successful in improving neonatal survival [111]. Focus on modifiable risk factors will effect change, e.g., age-at-first freshening, body condition score pre calving, gestational nutrition, dry period length, sire choice, preventing prolonged gestation, disease control, calving management and investigation of perinatal losses [20]. Less progress will be made with less modifiable risk factors, e.g., breed, parity, season of calving, cow:labour unit ratio, foetal gender and twin pregnancies.

### 3.7. It’s a Problem Farm Problem

Once the stakeholders decide to act to improve neonatal survival they need to focus effort. The skewed distribution of perinatal loss rates means that inter-herd variation in loss must be identifiable. Once problem herds, for example those in the top quartile of loss rates, can be identified (through targeted surveillance) they must be investigated through farm-level collection of a herd history and examination of records, examination of the pregnant animals, and assessment of calving management and collation of necropsy and laboratory findings. These are multi-disciplinary tasks involving data recording organisations, practising and laboratory veterinarians and extension professionals.

## 4. Conclusions

High bovine perinatal mortality rates remain an international welfare problem though this is often not recognised at national or at farm-level. Improvement in calf survival rates is dependent upon re-prioritization of this problem relative to other animal health and welfare issues and creation of awareness of this prioritization. Once the problem is recognised action needs to be taken at national and at farm levels, specifically on problem farms. Data recording, research, breeding, veterinary, extension and farmer organisations all have a role to play in improving bovine neonatal survival and hence improving animal welfare in the future. Ultimately improvements in welfare will be achieved by the aggregation of marginal gains across each of these inter-related disciplines.

## References

[1] Mee J.F. (2008). Newborn dairy calf management. Vet. Clin. North Am. Food Anim. Pract..

[2] Garry F., Benson G.J., Rollin B.E. (2004). An overview of animal welfare in the U.S. dairy industry. The Well-Being of Farm Animals: Challenges and Solutions.

[3] Uetake K. (2013). Newborn calf welfare: A review focusing on mortality rates. Anim. Sci. J..

[4] Nyman A., Lindberg A., Sandgren C. (2011). Can pre-collected register data be used to identify dairy herds with good cattle welfare?. Acta Vet. Scand..

[5] Smith R.F., Jones E.R., Waterman M. (2012). Tesco livestock code of practice. Cattle Pract..

[6] Sandgren C., Lindberg A., Keeling L. (2009). Using a national dairy database to identify herds with poor welfare. Anim. Welf..

[7] Vasseur E., Borderas F., Cue R., Lefebvre D., Pellerin D., Rushen J., Wade K., de Passille A. (2010). A survey of dairy calf management practices in Canada that affect animal welfare. J. Dairy Sci..

[8] Laven R., Huxley J., Whay H., Stafford K. (2009). Results of a survey of attitudes of dairy veterinarians in New Zealand regarding painful procedures and conditions in cattle. N. Z. Vet. J..

[9] Huxley J., Whay H. (2006). Current attitudes of cattle practitioners to pain and the use of analgesics in cattle. Vet Rec..

[10] Mellor D., Stafford K. (2004). Animal welfare implications of neonatal mortality and morbidity in farm animals. Vet. J..

[11] Brusseau R. (2006). Developmental perspectives: Is the fetus conscious?. Int. Anaest. Clin..

[12] More S., McKenzie K., O’Flaherty J., Doherty M., Cromie A., Magan M. (2010). Setting priorities for non-regulatory animal health in Ireland: Results from an expert Policy Delphi study and a farmer priority identification survey. Prev. Vet. Med..

[13] Steinbock A., Näsholm B., Berglund K., Johansson, Philipsson J. (2003). Genetic effects on stillbirth and calving difficulty in Swedish Holsteins at first and second calving. J. Dairy Sci..

[14] Hansen M., Misztal I., Lund M.S., Pedersen J., Christensen L.G. (2004). Undesired phenotypic and genetic trend for stillbirth in Danish Holsteins. J. Dairy Sci..

[15] van Pelt M., de Jong G. (2011). Genetic evaluation for direct and maternal livability in The Netherlands. Interbull Bull..

[16] Silva del Rio N., Stewart S., Rapnicki P., Chang Y.M., Fricke P.M. (2007). An observational analysis of twin births, calf sex ratio, and calf mortality in Holstein dairy cattle. J. Dairy Sci..

[17] Brickell J., Pollott G., Clempson A., Otter N., Wathes D. (2010). Polymorphisims in the bovine leptin gene associated with perinatal mortality in Holstein-Friesian heifers. J. Dairy Sci..

[18] Heringstad B., Chang Y., Svendsen M., Gianola D. (2007). Genetic analysis of calving difficulty and stillbirth in Norwegian Red cows. J. Dairy Sci..

[19] Vernooy E., Leslie K., Kelton D., Hand K., Muir B., Duffield T. (2007). Management risk factors associated with stillbirth. Proc. AABP.

[20] Mee J.F., Sanchez-Miguel C., Doherty M. (2013). Influence of modifiable risk factors on the incidence of stillbirth/perinatal mortality in dairy cattle. Vet. J..

[21] Szenci O., Nagy K., Tackas L., Madl L., Bajcsy C. (2012). Farm personnel management as a risk factor for stillbirth in Hungarian Holstein-Friesian dairy farms. Magyar Allat. Lapja.

[22] Heins B., Hansen L., Seykora A. (2006). Calving difficulty and stillbirths of pure Holsteins *versus* crossbreeds of Holstein with Normande, Montbelairde and Scandinavian Red. J. Dairy Sci..

[23] Mee J.F. (2013). Genetic selection impacts neonatal calf losses in Holsteins. Progr. Dairyman.

[24] Mee J.F., Berry D., Cromie A.R. (2008). Prevalance of, and risk factors associated with, neonatal calf mortality in pasture based Holstein-Friesian cows. Animal.

[25] Mee J.F., Grant J., Sanchez-Miguel C., Doherty M. (2013). Pre-calving and calving management practices in dairy herds with a history of high or low bovine perinatal mortality. Animals.

[26] McClintock S. (2004). A genetic evaluation of dystocia in Australian Holstein-Friesian cattle. Ph.D. Thesis.

[27] Egger-Danner C., Furst C., Mayerhofer M., Rain C. (2010). ZuchtData Jahresbericht 2010.

[28] Jamrozik J., Fatehi J., Kistemaker J., Schaeffer L. (2005). Estimates of genetic parameters for Canadian Holstein female reproduction traits. J. Dairy. Sci..

[29] Hoedemaker M., Ruddat I., Teltscher M., Essemeyer K., Kreinenbrock L. (2010). Influence of animal, herd, and management factors on perinatal mortality in dairy cattle—A survey in Thuringia, Germany. Berl. und Munch. Tierarzt. Wschnschrift..

[30] Benjaminsson B.H. (2007). Prenatal death in Icelandic cattle. Acta Vet Scand..

[31] Verma A., Gupta K. (2002). Genetic and non-genetic factors on incidence of abnormal calvings and components of replacement rate. SARAS J. Livest Poult. Prod..

[32] Bar D., Ezra E. (2005). Effects of common calving diseases on milk production in high yielding dairy cows. Israel J. Vet. Med..

[33] Ansari-Lari M. (2007). Study of perinatal mortality and dystocia in dairy cows in Fars Province, Southern Iran. Int. J. Dairy Sci..

[34] Raboisson D., Delor F., Cahuzac E., Gendre C., Sans P., Allaire G. (2013). Perinatal, neonatal, and rearing period mortality of dairy calves and replacement heifers in France. J. Dairy Sci..

[35] Pryce J.E., Harris B.L., Sim S., McPherson A.W. (2006). Genetics of stillbirth in dairy calves. Proc. N. Z. Soc. Anim. Prod..

[36] Piwczynski D., Nogalski Z., Sitowska B. (2013). Statistical modeling of calving ease and stillbirths in dairy cattle using the classification tree technique. Livest. Sci..

[37] Steinbock L., Johansson K., Nasholm A., Berglund B., Philipsson J. (2006). Calving difficulty in Swedish Red cattle at first and second calving. Acta Agric. Scand A.

[38] Bleul U. (2011). Risk factors and rates of perinatal and postnatal mortality in cattle in Switzerland. Livest. Sci..

[39] Brickell J.S., McGowan M., Pfeiffer D., Wathes D.C. (2009). Mortality in Holstein-Friesian calves and replacement heifers, in relation to body weight and IGF-I concentration, on 19 farms in England. Animal.

[40] Hansen M., Lund M., Pedersen J., Christensen L. (2004). Genetic parameters for stillbirth in Danish Holstein cows using a Bayesian threshold model. J. Dairy Sci..

[41] Meyer C.L., Berger P.J., Koehler K.J. (2000). Interactions among factors affecting stillbirths in Holstein cattle in the United States. J. Dairy Sci..

[42] McParland S., Kearney F., Rath M., Berry D. (2007). Inbreeding effects on milk production, calving performance, fertility, and conformation in Irish Holstein-Friesians. J. Dairy Sci..

[43] Johanson J.M., Berger P.J. (2003). Birth weight as a predictor of calving ease and neonatal mortality in Holstein cattle. J. Dairy Sci..

[44] Gulliksen S.M., Lie K.I., Loken T., Osteras O. (2009). Calf mortality in Norwegian dairy herds. J. Dairy Sci..

[45] Enjalbert F., Lebreton P., Salat O. (2006). Effects of copper, zinc and selenium status on performance and health in commercial dairy and beef herds: Retrospective study. J. Anim. Physiol. Anim. Nutr..

[46] Murray R.D., Williams A.J., Sheldon I.M. (2008). Field investigation of perinatal mortality in Friesian cattle associated with myocardial degeneration and necrosis. Reprod. Dom. Anim..

[47] McCoy M.A., Smyth J.A., Ellis W.A., Kennedy D.G. (1997). Experimental reproduction of iodine deficiency in cattle. Vet. Rec..

[48] Mee J.F., Rogers P.A.M., O’Farrell K.J. (1995). Effect of precalving feeding of a mineral-vitamin supplement before calving on the calving performance of a trace element-deficient dairy herd. Vet. Rec..

[49] Chassagne M., Barnouin J., Chacornac J.P. (1999). Risk factors for stillbirth in Holstein heifers under field conditions in France: A prospective survey. Theriogenology.

[50] Mee J.F., Garnsworthy P., Wiseman J. (2013). Impacts of nutrition pre-calving on periparturient dairy cow health and neonatal calf health. Recent Advances in Animal Nutrition.

[51] Gundelach Y., Essemeyer K., Teltscher M., Hoedemaker M. (2009). Risk factors for perinatal mortality in dairy cattle: Cow and foetal factors, calving process. Theriogenology.

[52] Berglund B., Steinbock L., Elvander M. (2003). Causes of stillbirth and time of death in Swedish Holstein calves examined post mortem. Acta Vet Scand..

[53] Mee J.F. The current causes of bovine perinatal mortality—A new diagnostic perspective. Proceedings of 26th World Buiatrics Congress.

[54] Mee J.F., Sanchez-Miguel C., Doherty M. (2013). An international Delphi study of the causes of death and the criteria used to assign cause of death in bovine perinatal mortality. Reprod. Dom. Anim..

[55] Stubbings D.P. (1992). Observations on sterile sequential autolysis in the bovine foetus. In. Proceedings of BCVA 1991–92.

[56] Dillman R.C., Dennis S.M. (1976). Sequential sterile autolysis in the ovine fetus: Macroscopic changes. Am. J. Vet. Res..

[57] Dillman R.C., Dennis S.M. (1979). Sequential sterile autolysis in the ovine fetus: Microscopic changes. Am. J. Vet. Res..

[58] McFarlane D. (1965). Perinatal lamb losses I. An autopsy method for the investigation of perinatal losses. N. Z. Vet. J..

[59] Johnston N.E., Condron R., Forsyth W.M., Nugent G.F. Profile of post-mortem changes in piglets which die before birth. Proceedings of International Pig Veterinary Society, 10th Congress.

[60] Genest D.R., Singer D.B. (1992). Estimating the time of death in stillborn foetuses: III. External fetal examination; A study of 86 stillborns. Obstet. Gynecol..

[61] Genest D.R., Williams M.A., Greene M.F. (1992). Estimating the time of death in stillborn foetuses: I. Histologic evaluation of fetal organs; an autopsy study of 150 stillborns. Obstet. Gynecol..

[62] Smyth J., Goodall E., McCoy M., Ellis W. (1996). Stillbirth/perinatal weak calf syndrome: A study of calves with an abnormal thyroid gland. Vet. Rec..

[63] Waldner C., Kennedy R., Rosengren L., Pollock C., Clark E. (2010). Gross postmortem and histologic examination findings from abortion losses and calf mortalities in Western Canadian beef herds. Can. Vet. J..

[64] Syrjala P., Anttila M., Dillard K., Fossi M., Collin K., Nylund M., Autio T. (2007). Causes of bovine abortion, stillbirth and neonatal death in Finland 1999–2006. Acta Vet. Scand..

[65] Siguroarson S., Vioarsson H., Jonsson M. (2008). Orsakir kalfadauoa um buro hja fyrsta kalfs kvigum. Orsakir kalfadauoa hja fyrsta kalfs kvigum. Syyrsla um rannosoknir 2006–2008. Landbunaoarhaaskoll Islands Rit Lbnl.

[66] Mee J.F., Hernandez C.T. (2013). A novel, illustrated classification system to define the causes of bovine perinatal mortality internationally. Dairy Cows. Reproduction, Nutritional Management and Diseases.

[67] Muskens J. Stillbirth in heifers: Experiences in The Netherlands. Proceedings of 25th World Buiatrics Congress.

[68] Schefers J. (2009). Why stillborns are still born. Hoard’s Dairyman.

[69] Mee J.F., Szenci O. (2012). Selected pathological causes of bovine stillbirth illustrated with photographic images. Mag. Allat. Lapja.

[70] Dufty J.H., Sloss V. (1977). Anoxia in the bovine fetus. Aust. Vet. J..

[71] Mee J.F., Kenneally J. Congenital intestinal atresia—More common than we think?. Proceedings of 20th International Congress of Hungarian Association for Buiatrics.

[72] Sluijter F., Zimmer G., Wouda W. (1990). Weak calf syndrome. Vet. Rec..

[73] MacDiarmid S. (1983). Induction of parturition in cattle using corticosteroids: A review. Anim. Breed. Abstr..

[74] Mee J.F. (1991). Premature expulsion of the placenta and bovine neonatal mortality. Vet. Rec..

[75] Smyth J., McNamee P., Kennedy D., McCullough S., Logan E., Ellis W. (1992). Stillbirth/perinatal weak calf syndrome: Preliminary pathological, microbiological and biochemical findings. Vet. Rec..

[76] Guyot H. (2008). Contribution to diagnosis and correction of iodine and selenium deficiencies in cattle. DSV Thesis.

[77] Gustafsson H., Kindahl H., Berglund B. (2007). Stillbirths in Holstein heifers—Some results from Swedish research. Acta Vet Scand..

[78] Kornmatitsuk B., Dahl E., Ropstad E., Beckers J.F., Gustafsson H., Kindahl H. (2004). Endocrine profiles, haematology and pregnancy outcomes of late pregnant Holstein dairy heifers sired by bulls giving a high or low incidence of stillbirth. Acta Vet. Scand..

[79] Stefaniak T., Jawor P., Bednarski M., Twardon J., Bajzert J., Sitnik O. Weak calf syndrome caused by chorioamnionitis and fetal inflammatory response syndrome (FIRS). Proceedings of 26th World Buiatrics Congress.

[80] Mee J.F. (2013). Explaining unexplained bovine stillbirth: How to deal with ‘farm blindness’. Vet. J..

[81] Gulliksen S., Lie K., Osteras O. (2009). Calf health monitoring in Norwegian dairy herds. J. Dairy Sci..

[82] Vasseur E., Rushen J., de Passille A.M., Lefebvre D., Pellerin D. (2010). An advisory tool to improve management practices affecting calf and heifer welfare on dairy farms. J. Dairy Sci..

[83] Mork M., Lindberg A., Alenius S., Vagsholm I., Egenvall A. (2009). Comparison between dairy cow disease incidence in data registered by farmers and in data from a disease-recording system based on veterinary reporting. Anim. Reprod. Sci..

[84] Svensson C., Sandgren C., Carlsson J., Svensson T., Tornquist M., de Verdier K. (2008). There is a need for veterinary involvement in calf rearing, but how will they gain admission?. Sven. Vet..

[85] Henderson L., Miglior F., Sewalem A., Kelton D., Robinson A., Leslie K. (2011). Estimation of genetic parameters for measures of calf survival in a population of Holstein heifer calves from a heifer-raising facility in New York State. J. Dairy Sci..

[86] O’Kane B. The stabiliser cattle breeding system. Proceedings of The Cattle Association of Veterinary Ireland Annual Conference.

[87] Lorenz I., Mee J.F., Earley B., More S. (2011). Calf health from birth to weaning. I. General aspects of disease prevention. Irish Vet. J..

[88] Seidenspinner T., Bennewitz J., Reinhardt F., Thaller G. Need for sharp phenotype in QTL detection for calving traits in dairy cattle. Proceedings of 58th Annual Meeting of the European. Association for Animal Production.

[89] Wiggans G.R., Cole J.B., Thornton L.L.M. (2008). Multiparity evaluation of calving ease and stillbirth with separate genetic effects by parity. J. Dairy Sci..

[90] Coffey M.P., Roughsedge T., Simm G. (2008). Tools for genetic improvement of maternal traits in beef cattle. Cattle Pract..

[91] Swalve H.H., Schafberg R., Rosner F. Genetic parameters for categories of stillbirth in dairy cattle. Proceedings of 8th World Congress on Genetics Applied to Livestock Production, Belo Horizonte.

[92] Dwyer C.M., Lawrence A.B. (2005). Frequency and cost of human intervention at lambing: An interbreed comparison. Vet. Rec..

[93] Miedema H., Cockram M., Dwyer C., Mcarae A. (2011). Changes in the behaviour of dairy cows during the 24 h before normal calving compared with behaviour during late pregnancy. Appl. Anim. Behav. Sci..

[94] Bahr C., Kaufmann O., Scheibe K., Cox S. (2007). Sensor based analysis and modelling of moving and resting behaviour in suckler cows before, during and after calving. Precision Livestock Farming ’07.

[95] Maltz E., Antler A., Cox S. (2007). A practical way to detect approaching calving of the dairy cow by a behaviour sensor. Precision Livestock Farming ’07.

[96] Cangar O., Leroy T., Guarino M., Vranken E., Fallon R.J., Lenehan J.J., Mee J.F., Berckmans D., Cox S. (2007). Model-based calving monitor using real time image analysis. Precision Livestock Farming ’07.

[97] Streyl D., Sautrer C., Braunert A., Lange D., Weber F., Zerbe H. (2011). Establishment of a standard operating procedure for predicting the time of calving in cattle. J. Vet. Sci..

[98] Shah K.D., Nakao T., Kubota H. (2006). Plasma estrone sulphate (E1S) and estradiol-17 beta (E2beta) profiles during pregnancy and their relationship with the relaxation of sacrosciatic ligament, and prediction of calving time in Holstein-Friesian cattle. Anim. Reprod. Sci..

[99] Bleul U., Schwantag S., Kahn W. (2008). Blood gas analysis of bovine fetal capillary blood during stage II labor. Theriogenology.

[100] Jenkin G., Ward J., Hooper S.B., O’Conner A.E., de Kretser D.M., Wallace E.M. (2001). Feto-placental hypoxemia regulates the release of fetal activin A and progesterone E2. Endocrinology.

[101] Bleul U., Kahn W. (2008). Monitoring the bovine fetus during stage II of parturition using pulse oximetry. Theriogenology.

[102] Bleul U., Lejeune B., Schwantag S., Kahn W. (2007). Ultrasonic transit-time measurement of blood flow in the umbilical arteries and veins in the bovine fetus during stage II of labor. Theriogenology.

[103] Uystepruyst C.H., Reinhold P., Coghe J., Bureau F., Lekeux P. (2000). Mechanics of the respiratory system in healthy newborn calves using impulse oscillometry. Res. Vet. Sci..

[104] Becker M., Tsousis G., Lupke M., Goblet F., Heun C., Seifert H., Bollwein H. (2010). Extraction forces in bovine obstetrics: An *in vitro* study investigating alternate ans simultaneous traction modes. Theriogenology.

[105] Tsousis G., Becker M., Lupke M., Goblet F., Heun C., Seifert H., Bollwein H. (2011). Extraction methods in bovine obstetrics: Comparison of the demanded energy and importance of calf and traction method in the variance of force and energy. Theriogenology.

[106] Van Engelen E., Breeveld-Dwarkasing V.N., Taverne M.A., van der Weijden G.C., Rutten V.P. (2008). MMP-2 expression precedes the final ripening process of the bovine cervix. Molecular Reprod. Devel..

[107] Van Engelen E., Taverne M.A.M., Everts M.E., van der Weijden G.C., Doornenbal A., Breeveld-Dwarkasing V.N.A. (2007). EMG activity of the muscular and stromal layer of the cervix in relation to EMG activity of the myometrium and cervical dilatation in PGF2alpha induced parturition in the cow. Theriogenology.

[108] Hirsbrunner G., Zanolari P., Althaus H., Husler J., Steiner A. (2007). Influence of prostaglandin E2 on parturition in cattle. Vet. Rec..

[109] Mainau E., Manteca X. (2011). Pain and discomfort caused by parturition in cows and sows. Appl. Anim. Behav. Sci..

[110] Mee J.F., Dahnof L.T. (2009). Bovine perinatology: Current understanding and future developments. Animal Reproduction: New Research Developments.

[111] Schuenemann G.M., Bas S., Gordon E., Workman J. (2011). Dairy calving management: Assessment of a comprehensive program for dairy personnel. J. Dairy Sci..

